# Validation of asthma recording in electronic health records: a systematic review

**DOI:** 10.2147/CLEP.S143718

**Published:** 2017-12-01

**Authors:** Francis Nissen, Jennifer K Quint, Samantha Wilkinson, Hana Mullerova, Liam Smeeth, Ian J Douglas

**Affiliations:** 1 Department of Non-Communicable Disease Epidemiology, London School of Hygiene and Tropical Medicine, London, UK; 2 National Heart and Lung Institute, Imperial College, London, UK; 3 RWD & Epidemiology, GSK R&D, Uxbridge, UK

**Keywords:** sensitivity, specificity, PPV, NPV, database, validity, epidemiology

## Abstract

**Objective:**

To describe the methods used to validate asthma diagnoses in electronic health records and summarize the results of the validation studies.

**Background:**

Electronic health records are increasingly being used for research on asthma to inform health services and health policy. Validation of the recording of asthma diagnoses in electronic health records is essential to use these databases for credible epidemiological asthma research.

**Methods:**

We searched EMBASE and MEDLINE databases for studies that validated asthma diagnoses detected in electronic health records up to October 2016. Two reviewers independently assessed the full text against the predetermined inclusion criteria. Key data including author, year, data source, case definitions, reference standard, and validation statistics (including sensitivity, specificity, positive predictive value [PPV], and negative predictive value [NPV]) were summarized in two tables.

**Results:**

Thirteen studies met the inclusion criteria. Most studies demonstrated a high validity using at least one case definition (PPV >80%). Ten studies used a manual validation as the reference standard; each had at least one case definition with a PPV of at least 63%, up to 100%. We also found two studies using a second independent database to validate asthma diagnoses. The PPVs of the best performing case definitions ranged from 46% to 58%. We found one study which used a questionnaire as the reference standard to validate a database case definition; the PPV of the case definition algorithm in this study was 89%.

**Conclusion:**

Attaining high PPVs (>80%) is possible using each of the discussed validation methods. Identifying asthma cases in electronic health records is possible with high sensitivity, specificity or PPV, by combining multiple data sources, or by focusing on specific test measures. Studies testing a range of case definitions show wide variation in the validity of each definition, suggesting this may be important for obtaining asthma definitions with optimal validity.

## Background

Asthma is one of the most common chronic diseases, and its core symptoms are cough, wheeze, breathlessness, and chest tightness.^1^ There is no cure, but with the right treatment, symptoms ranging from mild attacks to severe and life-threatening exacerbations^2^ can be managed.^1^ Despite this, a sizeable percentage of asthma patients are poorly controlled.^3^,^4^

Electronic health records (EHRs) have been widely adopted, which allows for the construction of large population-based patient databases. The availability of these routinely generated longitudinal records for research has greatly increased over the last decades.^5^ However, the accuracy of diagnoses recorded in these large databases may be low, which would introduce bias into studies using the data. Unless the data are validated for research, the quality of studies generated from EHRs may be debatable.^6^–^9^ Furthermore, the validity of different disease definitions is not always the same in a given dataset. Some diseases (such as asthma) might be coded using less specific symptoms, whereas the validity of diagnoses with very specific symptoms (such as tension pneumothorax) is likely to be better.

EHRs predominantly store information about diagnoses as clinical codes. A single code, or a case definition consisting of multiple codes (with or without additional information such as tests or prescribing) can be used to retrieve records from EHRs, and additional restrictions can be applied such as age or exclusion of other diseases.^9^,^10^ Validity of coding is generally assessed by comparing a code (or algorithm) to 1) the diagnosis as verified by the treating physician either by manual review of the chart notes or in clinic, 2) a reference standard such as another linked dataset, or 3) a patient questionnaire.^10^ A previous systematic review by Sharifi et al reviewed validation methods to capture acute bronchospasm in administrative or claims data;^11^ this review identified two validation studies of bronchospasm codes.^12^,^13^ However, the study was limited to administrative and claims databases, from the United States and Canada. Al Sallakh et al explored approaches to defining asthma or assessing asthma outcomes using EHR-derived data in the recent literature (calendar years 2014 and 2015) and examined the clarity of reporting.^14^ This systematic review focuses on how asthma was defined and does not include an overview of test measures or validation statistics.

There is currently no consensus on approaches to defining asthma or assessing asthma outcomes using EHR-derived data. We explored these approaches in the recent literature and examined the clarity of reporting.

## Research objective

The primary objectives of this systematic review are to provide an overview of the methods used in the literature for validating asthma diagnosis in EHRs, and the corresponding estimates of the validation test measures.

## Methods

**Figure 1:**
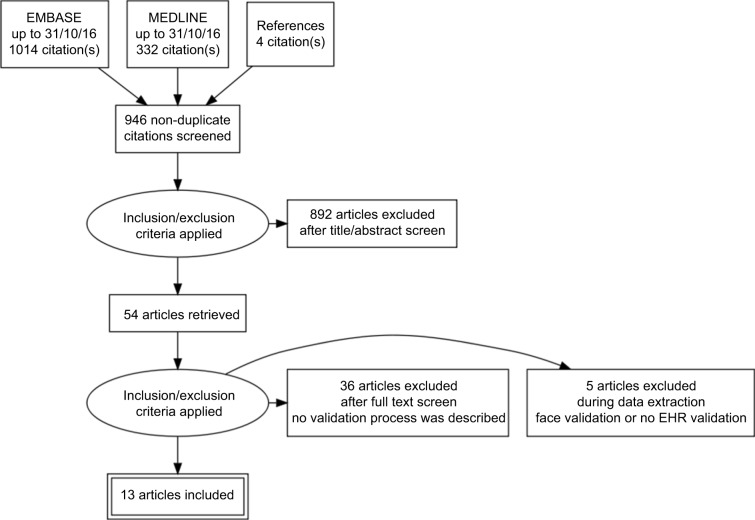
Study screening process: PRISMA flow diagram. **Note:** Reproduced from Moher D, Liberati A, Tetzlaff J, Altman DG, PRISMA Group. Preferred reporting items for systematic Reviews and meta-analyses: the PRISMA statement. *BMJ*. 2009;339:b2535.^37^ **Abbreviation:** EHR, electronic health record.

The methods are described in detail in the study protocol.^15^ We searched MEDLINE and EMBASE up to October 2016 for relevant articles. Our search strategy was composed of the following sets of terms: 1) electronic health records or databases AND 2) validity or validation or case definition or algorithm or sensitivity or specificity or positive predictive value or negative predictive value AND 3) the medical subject heading terms for asthma. Reference lists of articles of interest were reviewed to add potential additional studies in which a validation of asthma diagnosis was done. The PRISMA flow diagram can be found in Figure 1 and the search strategy can be found in the supplementary material. We considered any type of observational study design that used EHR to validate the recording of a diagnosis of asthma. In addition, we required a clear case definition to define asthma from EHR, including a description of the validation of said case definition through at least one test measure (sensitivity, specificity, positive predictive value [PPV] or negative predictive value [NPV]). Two investigators (FN and SW) separately assessed the abstracts and full text of each potential study against our inclusion criteria; disagreements were resolved through a third investigator or by discussion to reach consensus. The first author extracted all relevant data regarding methodologic elements of included studies; author, year of publication, country, time period, date, data source, population, case characteristics, clinical events, algorithms, reference standard, and validation statistics. Bias was assessed using QUADAS-2 tailored to this specific review.^16^

The questions of interest for this systematic review are: 1) which EHR databases were used to obtain information on the diagnosis of asthma? 2) Which case definitions, algorithms or codes were used to define an asthma diagnosis? 3) How were the diagnostic criteria applied to the data sources and which other approaches have been used to validate a case definition algorithm? and 4) What are the estimates for the PPV, NPV, specificity, and sensitivity for a diagnosis of asthma in an EHR?

### Inclusion criteria

Any type of observational study design which validated the recording of an asthma diagnosis in EHR was considered. Articles were only considered if published in English and published before October 2016 without any specific start date. Within the databases, we considered asthma diagnoses based on both structured data (such as laboratory results and prescriptions) and unstructured data (such as spirometry results). We required the validation case definitions to be compared to an external reference standard, such as a manual review, questionnaires (completed by the patient or their physician) or an independent second database. We included case definitions formed of single codes, those requiring multiple case characteristics, and case definitions generated by natural language processing (NLP) and/or machine-learning.

### Exclusion criteria

EHRs are a digital reflection of the key facts a health care provider needs to record in order to facilitate ongoing and potentially complex clinical care. By contrast, the main purpose of administrative claims data is administration of reimbursements to health care providers for their services. This systematic review included only studies from EHRs, as the quality measures between the two types of data can be markedly different; studies using administrative claims data were excluded. Studies involving pharmacovigilance databases (signal detection or spontaneous reporting), studies without validation of asthma recording, and conference abstracts were excluded.^17^,^18^

### Data synthesis

Studies and study data were managed using EndNote and Microsoft Excel, respectively.

The methods for validation of asthma recording in the included studies were outlined in a narrative synthesis. In addition, Table 1 summarizes the methods and Table 2 describes the results, consisting of the recorded PPV, NPV, sensitivity, and specificity of the included studies.

### Dissemination and ethics

This study is a synthesis of previously published studies, so no ethical approval is required. The protocol was registered in the PROSPERO database with registration number CRD42016041798, and the protocol has been published.^15^ Results from this systematic review can be used to study outcome research on asthma and can be used to identify case definitions for asthma.

## Results

In total, 1,346 titles were found in the EMBASE and MED-LINE databases, of which 946 were non-duplicates. Of those, 54 articles were reviewed in full text and we found 13 articles that contained a validation process of asthma diagnosis that met all eligibility criteria. Characteristics of the 13 included studies ordered by year of publication are summarized in Table 1, and the study results are displayed in Table 2. The asthma prevalence necessary for the interpretation of PPVs and NPVs is presented in Table 1, where available.

The reference standard used to validate the asthma diagnosis in the EHRs differed between the studies: ten studies used manual validation by a clinician, two studies compared the studied records to independent linked databases and one study used patient questionnaires. The test measures also differ between the different papers, encompassing sensitivity, specificity, PPV, and NPV. We focus on 13 studies in this review, ordered by reference standard used and by date of publication. Bias assessment results using QUADAS-2 are presented in Table 3.

### Manual validation

We found ten studies that used a manual validation as the reference standard. All studies had at least one case definition algorithm with a PPV of at least 63%. Where other measurements could be calculated, the studies had at least one case definition with a sensitivity of at least 85%, specificity of at least 92%, and NPV of at least 94%. Within this group, four studies used case definition algorithms generated by machine learning. Five studies included only children, while two studies included only persons older than 16 years.

Xi et al tested a variety of EHR search algorithms based on two large academic primary care clinics in Hamilton, Canada.^19^ The reference standard consisted of a physician chart review-based diagnosis. The eight case definitions are presented in Table 1, and their PPVs in Table 2. The algorithm searching for patients who had asthma in their patient profile or had an asthma billing code was the most accurate with a sensitivity of 90% (95% CI [87% to 93%]) and a specificity of 84% (95% CI [80% to 88%]).

Engelkes et al undertook a study to determine the validity of case definitions generated by machine learning to define asthma cases, based on a previous study be Afzal et al.^20^,^21^ Originating from a large Dutch general practitioner (GP) database, the authors manually reviewed 22,699 potential asthma cases. Among those, 14,303 asthma cases were found, which resulted in a PPV of 63%.

The study by Afzal et al uses the same dataset and machine-learning algorithm for definite and potential asthma cases as the study by Engelkes et al.^20^,^21^ Clinicians manually validated 5,032 potential asthma cases identified by a broad search algorithm out of 63,618 patients. This training set was used for the machine-learning algorithm. The test measures are measuring the validity of the machine-learning algorithm within the smaller population, not of the broad search algorithm. The PPV, sensitivity, and specificity for three case definition algorithms (definite cases; definite and probable cases; definite, probable, and doubtful cases) were calculated. The PPVs range from 57% for all definite, probable, and doubtful asthma cases to 82% for only the definite asthma cases.

Dexheimer et al evaluated a computerized asthma detection system in an urban, tertiary care pediatric emergency department in a 3-month prospective, randomized controlled trial in 2009.^22^ A Bayesian network system screened all emergency department patients for acute asthma. The system identified 1,100 patients with asthma exacerbations, of which 704 were confirmed by a pediatric emergency care physician within 3 days of the visit. The PPV for the Bayesian network system was 65%.

Wu et al evaluated the accuracy of a computational approach to asthma ascertainment. The authors developed an NLP system for extracting predetermined asthma from free text in EHRs.^23^ Manual chart review by a clinician was the reference standard. The patient group consisted of 112 children younger than 4 years. The NLP-generated case definition algorithms had a sensitivity of 85%, specificity of 97%, PPV of 88%, and an NPV of 95%. For comparison, the test measures of the ICD-9 asthma codes were calculated (sensitivity 31%, specificity 93%, PPV 57%, NPV 82%).

Kozyrskyj et al described the Study of Asthma, Genes and the Environment (SAGE). The study captures the longitudinal health care records of 16,320 children born in 1995 in Manitoba (Canada) and contains detailed information on early-life exposures in relationship to the development of asthma.^24^ Within the birth cohort, a nested case-control study with 723 children was partly created to confirm asthma status in children and these data were used to validate health care database measures of asthma. These 723 children were chosen by random sampling from the birth cohort; the parents of 288 children with and 435 without a parental report of asthma in the last 12 months agreed to participate. The reference standard for the validation consisted of pediatric allergist-diagnosed asthma, methacholine challenge tests, and skin tests. The PPV of asthma definitions varied from 90% to 94%, the sensitivity from 47% to 82%, and the specificity from 83% to 92%.

Pacheco et al constructed case definitions to identify asthmatic patients as cases, and healthy patients as controls using data from electronic medical records in the United States. This was done to identify asthma patients for future genome-wide association studies (GWAS). The case definitions consisted of a combination of diagnoses, medications, and smoking history.^25^ By applying stringent criteria, the study results show a PPV of 95% and an NPV of 96% for identification of asthma cases and controls, using clinician review as the reference standard. GWAS require a high specificity, PPV, and NPV. A high specificity was achieved but at the loss of 24% of the potential asthma cases.

Vollmer et al used the electronic databases of a large health maintenance organization to develop a case definition for defining prevalent asthma and to validate it against chart review.^26^ The data systems of this organization, the Kaiser Permanente Northwest Division consist of both EHR (inpatient data, emergency department data, EpicCare) and administrative data: “Outside claims database” and “The outpatient pharmacy system”. Table 2 presents the PPV of the eight different case definition algorithms to define asthma. The fourth case definition, based on a combination of an urgent care visit and the order of nebulizer treatment (N=25), had the highest PPV (100%), while the first case definition, based on non-urgent care visits, (N=4,460) had a PPV of 95% while identifying a much larger population.

Donahue et al sought to determine the reliability of identifying asthmatics through automated medical and pharmacy records. All adult members of the Harvard Pilgrim Health Care program who received an asthma diagnosis and at least one asthma drug between 1988 and 1991 were identified.^27^ The authors manually reviewed records of a random sample of 100 patients to validate the asthma diagnosis. The PPV of a coded asthma diagnosis was 86%.

Premaratne et al measured the validity of the string “asth” in the accident and emergency (A&E) department attendance diagnosis field for identifying patients with asthma-related conditions attending the A&E departments of two hospitals in the UK in 1995.^28^ A reception clerk entered the diagnosis field in a database at arrival in the A&E department. The reference standard was a confirmation of the asthma diagnosis by a clinical officer, or symptoms of asthma plus a history of asthma or bronchodilators given with improvement, or a previously diagnosed asthmatic with symptoms or prescribed asthma medication. An “attendance diagnosis” of asthma was excluded if there was a clear alternative diagnosis or sufficient other evidence to exclude asthma. The string “asth” in the attendance diagnosis field had a sensitivity of 80% (75%–86%) and a specificity of 97% (96%–98%) for a confirmation of asthma.

### Linked databases

Our search found two studies which used a second independent database to validate asthma diagnoses in the first database. The PPVs ranged from 46% to 58%.

Coulter et al^30^ compared repeat prescriptions for asthma, epilepsy, and thyroid disease with chronic disease registers stored on general practice computers in the early days of EHRs (1989). PPV of an asthma diagnosis on the register was 58% for asthma when using medication prescriptions as the reference standard.

Engeland et al evaluated the reliability of maternal disease registration (diabetes, asthma, and epilepsy) in the Medical Birth Registry of Norway (MBRN).^29^ The data they examined consisted of the EHRs of 108,489 pregnancies between April 2004 and January 2007. The reference standard was the prescriptions in the Norwegian Prescription Database (NorPD). The overall sensitivity of an asthma diagnosis in MBRN was 51% (49–52), but increasing when considering with a higher asthma treatment step in the NorPD. The sensitivity was 40% when considering records which only used inhaled selective beta-2-adrenoreceptor agonists (step1), while the sensitivity of asthma diagnosis in records with systemic drugs other than adrenergics for obstructive airway diseases was 73%.

### Questionnaires

There was only one study which used a questionnaire as the reference standard for database validation.

Ward et al aimed to determine the degree of under- or over-reporting of the diagnosis of asthma for patients aged 16–55 years in one large general practice in the UK.^31^ The case definition described in Table 1, (based on either codes, text strings or prescriptions) yielded 833 potential asthma cases and 831 age- and sex-matched controls from the GP database. A questionnaire validated for the detection of bronchial hyper-reactivity was sent to all asthma patients and their matched controls. Patients with a diagnosis of asthma and bronchial hyper-reactivity in the questionnaire were considered to have asthma. Evidence of asthma was sought for two groups: patients with asthma and without symptoms of bronchial hyper-reactivity, and controls with symptoms of bronchial hyper-reactivity. The results show an overall PPV of the case definition of 89%.

## Discussion

The main finding of this review is that case definitions and methods of asthma diagnosis validation vary widely across different EHR databases. This is evident in the diversity of databases used by the studies, such as primary care databases, combined EHR and administrative databases, or data from nested case-control studies within larger cohorts. Some databases originate from a single or a few health centers, while others span millions of patients. The source of the EHR databases (primary care, secondary care, and urgent care) influences the case definition of asthma and the way the validation is conducted. Patients seeking care for asthma symptoms will present differently in each setting, and the test measures might reflect this.

Case definitions are designed with different purposes in mind, and each of the studied test measures (sensitivity, specificity, PPV, and NPV) have different uses. A high sensitivity is needed to identify all asthma patients from a database, but if the aim is to exclude all records of patients who do not have asthma, a high specificity is more important.^32^ The PPV is the proportion of true positives among all positive results: the patients who have asthma in the examined database who also have asthma according to the reference standard. The NPV shows the proportion of true negatives among all negative results: patients that do not have asthma in the database who also do not have asthma according the reference standard. PPVs and NPVs are directly related to the prevalence of asthma in the population. The PPV will increase with rising prevalence; the NPV will decrease with rising prevalence assuming all other factors remain constant.

Studies, the main aim of which was not database validation, were able to demonstrate a high test measure to suit their specific needs (PPV, NPV, sensitivity or specificity greater than 80%). If this was not the case, their main study results (not including validation) would not be reliable, and thus potential studies with low validity of asthma diagnosis might not have been conducted or published. In contrast, studies with a main aim of validation of asthma in databases have a wider range of test measures depending on the case definition. The PPV in these studies range from 46%^29^ to 96%.^23^

Manual validation was the most common reference standard in the validation studies included in this systematic review. The computer-generated case definitions studied recently by Engelkes et al,^20^ Afzal et al,^21^ Dexheimer et al,^22^ and Wu et al^23^ provide ways to create algorithms with high sensitivities and specificities. The PPVs of these methods (whether a person identified as having an asthma diagnosis actually has asthma) might not be sufficient for all purposes (63%–82%). Preselected case definitions were used in five out of ten studies which manually validated the databases. The studies by Xi et al,^19^ Kozyrskyj et al,^24^ Pacheco et al,^25^ Vollmer et al,^26^ Donahue et al,^27^ and Premaratne et al^28^ used this approach and all report at least one case definition algorithm with a PPV above 85%. The best results arise when combining diagnostic data and prescription data.

Other studies by Engeland et al^29^ and Coulter et al^30^ used an external data source as reference standard. This approach needs two databases with near complete data, so their test measures are reliable on the quality and completeness of the two databases. It also requires that the validity of the reference standard is already known. However, they are much cheaper to carry out overall. Manual validation requires a considerable amount of time to complete, and questionnaires to hundreds of patients or clinicians can be expensive or unreliable. Coulter et al measured database completeness and integrity by studying different diseases including asthma. Their focus was not on asthma validation, but rather to check whether a digital database can be a valid alternative for analog registration.

Typical problems of validation studies are the lack of availability of a reliable reference standard and the interdependence of different data sources used for validation. There were four studies, not included in this review, which used face validity to compare the prevalence of asthma using a case definition to the general asthma prevalence. This method was not considered sufficiently exact for inclusion^33^–^36^ and by definition was unable to verify the validity of individual records.

The diagnosis of asthma can represent different conditions in different regions of the world. Thus, several authors used an inclusive strategy and many diagnosis codes in order to maximize sensitivity. Researchers must weigh the benefits of a case-finding algorithm with high sensitivity against the likely lower specificity and PPV, according to the purpose of their research. In future studies using predetermined case definitions, it may be of interest to evaluate the predictive value of a specific set of codes validated by chest physicians or GPs working in the health system the database originates from. This group may be more accurate when assigning the diagnosis, and the codes applied may yield a much higher predictive value than when evaluating the same group of codes assigned by all providers. The PPV, NPV, sensitivity, and specificity can differ greatly within a single study, as shown in the studies by Xi et al,^19^ Afzal et al,^21^ Kozyrskyj et al,^24^ and Vollmer et al.^26^ For this reason, the testing of multiple case definitions to obtain the algorithm with the highest test measure needed would be beneficial for future studies.

## Conclusion

Asthma validation studies using EHRs are very varied in their approach to the validation. This seems driven by the nature of the data and the reference standards used. Machine-learning methods of algorithm development allow for measuring all elements of validity. Different case definitions within a single data source have different validity, highlighting the importance of testing a range of case definitions.

## Strengths and limitations of this study

The review of validation of asthma diagnosis codes in EHRs informs selection of asthma definitions used by future studies and identify any gaps in quality and scope of validation studies. It also provides an overview of the case definitions and algorithms with their PPV, NPV, sensitivity or specificity.

Validated case definition algorithms are often very specific to the database they were developed in, limiting their generalizability.

Publication bias might be an issue as methods that do not find favorable results may be less likely to have been published.

## Data sharing statement

Study data are available on request to FN.

## Supplementary material

### Algorithm used for literature review

#### Asthma validation in electronic health records: a systematic review

##### MEDLINE

1. (validat* or verif*).mp. [mp=title, abstract, original title, name of substance word, subject heading word, keyword heading word, protocol supplementary concept word, rare disease supplementary concept word, unique identifier]
2. (PPV or PNV or NPV or “positive predictive value*” or “negative predictive value*” or “predictive positive value*” or “predictive negative value*” or “likelihood ratio” or precision or accuracy or “receiver operating characteristic*” or ROC or kappa).mp. [mp=title, abstract, original title, name of substance word, subject heading word, keyword heading word, protocol supplementary concept word, rare disease supplementary concept word, unique identifier]
3. Validation Studies/or validation.mp. or validation studies as topic/
4. (electronic* or digital* or computeri?ed or programmed or automated or database or data base).mp. [mp=title, abstract, original title, name of substance word, subject heading word, keyword heading word, protocol supplementary concept word, rare disease supplementary concept word, unique identifier]
5. asthma.mp. or Asthma/or Asthma, Occupational/or Asthma, Exercise-Induced/
6. Database Management Systems/
7. 1 or 2 or 3
8. 4 or 6
9. 5 and 7 and 8

##### EMBASE

1. (validat* or verif*).mp. [mp=title, abstract, heading word, drug trade name, original title, device manufacturer, drug manufacturer, device trade name, keyword]
2. validation.mp. or validation study/or validation process/
3. (sensitivity or specificity or “Sensitivity and Specificity”). mp. [mp=title, abstract, heading word, drug trade name, original title, device manufacturer, drug manufacturer, device trade name, keyword]
4. (PPV or PNV or NPV or “positive predictive value” or “predictive negative value” or “negative predictive value” or “likelihood ratio” or precision or accuracy or “receiver operating characteristic” or ROC or kappa).mp. [mp=title, abstract, heading word, drug trade name, original title, device manufacturer, drug manufacturer, device trade name, keyword, floating subheading]
5. (electronic* or digital* or computeri?ed or programmed or automated or database or data base).mp. [mp=title, abstract, heading word, drug trade name, original title, device manufacturer, drug manufacturer, device trade name, keyword]
6. mild persistent asthma/or nocturnal asthma/or experimental asthma/or moderate persistent asthma/or severe persistent asthma/or Asthma.mp. or exercise induced asthma/or occupational asthma/or intrinsic asthma/or asthma/or allergic asthma/or extrinsic asthma/or mild intermittent asthma/
7. 1 or 2 or 3 or 4
8. 5 and 6 and 7

## Figures and Tables

**Table 1:** Characteristics of studies with validated asthma algorithms

Author, year, country, (period)	Data source, population	Sample/case characteristics	Clinical event	Algorithm	Validation
**Manual validation**					
Xi et al,^19^ 2015 Canada	2 large academic primary care clinics Primary care	398 randomly selected patients 16 years and older	Asthma code COPD code Other respiratory condition code Other condition code	Search algorithms: 1. Asthma in disease registry 2. Billing code 3. Asthma in CPP 4. Asthma medications 5. Asthma in chart notes 6. Asthma in CPP OR billing code 493 7. Asthma in CPP OR billing code 493 (exclusion codes 491,492, and 496) 8. (Asthma in chart notes OR asthma medications) AND billing code 493 9. (Billing code 493 OR medications) AND asthma in chart notes 10. Billing diagnostic code 493 AND asthma in chart notes	Manual review
Engelkes et al,^20^ 2014 the Netherlands	ICPI: Dutch GP EHR Primary care	63,518 potential cases identified 22,699 cases after automated text validation Children aged 5–18	Definite, probable, and doubtful cases of asthma	Combination of ICPI communication codes, clinician codes, drug names and free text generated by a machine-learning algorithm (RIPPER)	22,699 cases manually validated, 14,303 asthma cases found
Afzal et al,^21^ 2013 the Netherlands January 2000–January 2012	ICPI: Dutch GP EHR Primary care	63,618 potential asthma cases identified, children aged 5–18	Definite, probable, and doubtful cases of asthma	Combination of ICPI communication codes, clinician codes, drug names and free text generated by a machine-learning algorithm (RIPPER)	5,032 patients manually validated by clinician
Dexheimer et al,^22^ 2013 United States	1 pediatric ED	15,163 assessed, 1,100 asthma patients all asthma patients (2–18 years) in a 3 month time window	Asthma code	Bayesian network system, previously used on claims data (Sanders)	Pediatric asthma/respiratory distress protocol filled in for identified patients
Wu et al,^23^ 2013, 2014 United States	Children enrolled in the Mayo Clinic sick-child daycare program, Secondary care	112 children younger than 4	ICD-9 codes Natural language	Natural language processing (logic) Natural language processing (machine learning)	Manual review by a clinician
Kozyrskyj et al,^24^ 2009 Canada	SAGE: birth cohort of 16,320 children born in 1995 in Manitoba, Canada Questionnaire in 2002 had 3,598 responses Manitoba’s health care registry records	723 children from the group with completed questionnaires 246 cases, 477 controls	Asthma	Database definitions in health care records	Pediatric allergist diagnosis of asthma
Pacheco et al,^25^ 2009 United States	NUgene Project Genome-wide association study	7,970 people with DNA samples, of which 521 had an asthma diagnosis	Asthma diagnosis	**Initial asthma cases algorithm:** Asthma diagnosis and asthma medication prescription on ≥1 visit AND no other chronic lung disease diagnosis on ≥2 visits AND no reported smoking history ≥10 years **Final asthma cases algorithm:** Asthma diagnosis on ≥1 visit AND asthma diagnosis or medication presciption on ≥1 other visit AND no other chronic lung disease diagnosis on ≥2 visits AND no reported smoking history ≥10 years **Initial asthma controls algorithm:** No diagnosis for any respiratory disease or cancer AND no prescription of any astha/COPD/iimmunodepressant medication AND no reported smoking history ≥10 years **Final asthma controls algorithm:** ≥2 visits with any asthma diagnosis or prescriptions AND no diagnosis for any respiratory disease or listed cancer AND no prescription of any asthma/COPD/immunodepressant medication AND no reported smoking history ≥10 years	Manual review of 100 cases for both algorithms
Vollmer et al,^26^ 2004 United States July 1998 to January 1999	KPNW, Epic, OSCAR, TOPS ED, secondary care	235,000 patients with continuous health plan eligibility aged 15–55 in January 1999 9,723 asthma patients identified	ICD-9 codes	**Health care utilization profiles used for validation study** 1. Four “high-probable” categories: → Two or more non-urgent care outpatient contacts for asthma → A single non-urgent contact and one or more ED or inpatient contact for asthma → Any Industrial Medicine visit for asthma → Any asthma visit and either of the two medication dispensing criteria 2. Single non-urgent outpatient visit only 3. Four or more β-agonists, with or without a nebulizer treatment order, but no asthma visits of any kind and no ICS dispensings 4. ED or urgent care visit for asthma and nebulizer treatment order, but no other medication criteria met and no other types of asthma visits 5. Hospitalization for asthma, but neither asthma medication criterion met and no outpatient asthma visits of any kind 6. ED or urgent care visit for asthma, but no other types of asthma visits and no asthma medication criteria met 7. Nebulizer treatment but no asthma visits of any kind and no other medication criteria met 8. All other cases	**Criteria used in medical records review Probable asthma** • Two or more asthma health care visits • A single visit for asthma with a chart notation indicating a prior history of asthma • A single health care visit for active symptoms of asthma (wheeze, cough, shortness of breath) • A single visit for an asthma exacerbation that responds to therapy, even if no prior history **Possible asthma** • Patient-reported history of asthma noted in chart, but no evidence of active asthma or treatment for asthma • An uncorroborated ED diagnosis of asthma • Diagnosis of “rule out asthma” with no clear resolution
Donahue et al,^27^ 1997 United States	Harvard Pilgrim Health Care (HPHC); Primary, secondary and emergency care	Random sample of 100 patients	Asthma code	Asthma diagnosis and asthma drug dispensing	Manual review by clinicians
Premaratne et al,^28^ 1997 United Kingdom 1994	Accident and EDs of two hospitals	All asthma patients January–March 1994 1,185 records, of which 209 did not have enough data	String containing “asth*”	String containing “asth*” in the free text records	**Affirmation of asthma diagnosis:** Final diagnosis of asthma by clinical officer OR symptoms of asthma and (history of asthma or bronchodilators given, with improvement) OR known asthmatic presented with symptoms or for medication **Rejection of asthma diagnosis:** Clear alternative diagnosis Sufficient other information to reject asthma diagnosis

**Comparison to an in dependent database**					

Engeland et al,^29^ 2009 Norway	MBRN: population-based birth registry, all births in Norway since 1967 (more than 2.3 million) NorPD: all dispensed prescriptions from January 2004 in Norway	108,489 pregnancies, of which 4,549 mothers were recorded as having asthma in MBRN	Asthma	Asthma diagnosis in MBRN	NorPD: asthma medication
Coulter et al,^30^ 1989 United Kingdom	7 general practices in the Oxford community health project 2,199 patients on medication Primary care	2,443 on digital register Bronchodilators, inhaled CS, prophylactic drugs	Asthma diagnosis	Asthma diagnosis on register	Manual review against the list of patients on long-term medication
**Comparison to a quiestionnaire**					
Ward et al,^31^ 2004 United Kingdom 1995–2004	GP Practice with 14,830 patients 83 1 controls, 587 responses Primary care	833 asthma patients, 659 responses 16–55 years on 1 October 1997	Asthma in GP database	One of the following criteria: 1. Read coded “asthma” diagnosis, H33 2. Attendances recorded on the asthma care screen 3. An intervention for asthma recorded 4. A textual entry “asthma” or “wheez” in the medical history 5. Inhaled steroids in the repeat prescriptions 6. Inhaled bronchodilators in the repeat prescriptions 7. Cromolyns in the repeat prescriptions	Questionnaire to determine bronchial hyperreactivity Cases: asthma in database Asthma diagnosis and bronchial hyperreactivity: considered positive Asthma diagnosis without bronchial hyperreactivity: further investigated in GP record Controls: bronchial hyperreactivity but no asthma diagnosis

**Abbreviations:** CPP, cumulative patient profile; ICPI, integrated primary care information database; GP, general practitioner; EHR, electronic health record; SAGE, Study of Asthma, Genes and the Environment; KPNW, Kaiser Permanente Northwest Division; OSCAR, outside claims database; TOPS, The outpatient pharmacy system; ED, emergency department; ICS, inhaled corticosteroids; MBRN, Medical Birth Registry of Norway; CS, corticosteroids; NorPD, Norwegian Prescription Database.

**Table 2:** Characteristics of studies with validated asthma algorithms

Author, year, country, prevalence	Algorithm	Sensitivity, 95% CI	Specificity, 95% CI	PPV, 95% CI	NPV, 95% CI	Prevalence
Manual validation						

Xi et al,^19^ 2015 Canada	1. Asthma in disease registry	7% (5–10)	99% (97–100)	67% (38–87)	73% (72–74)	8.1%
	2. Billing code	77% (75–83)	89.2% (86–92)	74% (67–80)	91% (88–94)	
	3. Asthma in CPP	63% (59–68)	92% (90–95)	76% (68–83)	87% (83–89)	
	4. Asthma medications	79% (75–83)	64% (59–68)	46% (41–50)	88% (84–92)	
	5. Asthma in chart notes	85% (81–88)	76% (72–80)	58% (52–63)	93% (89–95)	
	6. Asthma in CPP OR billing code 493	90% (87–93)	84% (80–88)	69% (63–74)	96% (93–97)	
	7. Asthma in CPP OR billing code 493 (exclusion codes 491, 492, and 496)	87% (83–90)	85% (82–89)	70% (63–76)	94% (91–96)	
	8. (Asthma in chart notes OR asthma medications) AND billing code 493	78% (74–82)	92% (89–95)	79% (72–85)	91% (88–94)	
	9. (Billing code 493 OR medications) AND asthma in chart note	84% (80–88)	84% (80–88)	67% (61–73)	93% (90–95)	
	10. Billing diagnostic code 493 AND asthma in chart notes	74% (70–78)	93% (91–96)	81% (73–87)	90% (87–93)	
Engelkes et al,^20^ 2014 Netherlands	Definite, probable and doubtful cases			63%		
Afzal et al,^21^ 2013 Netherlands	Definite asthma	98%	95%	66%		6%
	Definite + probable	96%	90%	82%		29%
	Definite, probable and doubtful cases	95%	67%	57%		32%
Dexheimer et al,^22^ 2013 United States	Algorithm constructed using a Bayesian network system			64%		7–10%
Wu et al,^23^ 2013, 2014 United States	ICD-9 codes	31	93	57	82	4–17%
	Natural language processing: logic	81	95	84	94	
	Natural language processing: machine learning	85	97	88	95	
Kozyrskyj et al,^24^ 2009 Canada	At least one asthma hospitalization, or two physician visits, or four prescription medications	47% (35–60)	92% (78–98)	91% (76–98)		11%
	At least one asthma hospitalization, or two physician visits, or two prescription medications	67% (54–78)	92% (78–98)	94% (82–99)		
	At least one asthma hospitalization, or one physician visit, or two prescription medications	77% (65–87)	92% (78–98)	94% (85–99)		
	At least one asthma hospitalization, or one physician visit, or two bronchodilators, or one controller medication	80% (69–89)	89% (74–97)	93% (83–98)		
	At least one asthma hospitalization, or one physician visit, or two bronchodilators, or one bronchodilator and ketotifen or an oral steroid, or one controller medication	80% (69–89)	89% (74–97)	93% (83–98)		
	At least one asthma hospitalization, or one physician visit, or one bronchodilator, or one controller medication	82% (70–90)	83% (67–94)	90% (79–96)		
Pacheco et al, 2009 United States	Initial algorithm	70% (60–78)	100%	100% (90–100)	77% (65–86)	7.2%
	Final algorithm	95% (84–99)	96% (87–99)	95% (84–99)	96% (87–99)	
Vollmer et al,^26^ 2004 United States	Algorithm 1: population of 4460			95%		4.1%
	Algorithm 2: population of 2334			90%		
	Algorithm 3: population of 545			70%		
	Algorithm 4: population of 25			100%		
	Algorithm 5: population of 11			50%		
	Algorithm 6: population of 721			80%		
	Algorithm 7: population of 99			27%		
	Algorithm 8: population of 1528			80%		
Donahue et al,^27^ 1997 United States	Asthma code and drug dispensing			86%		3%
Premaratne et al,^28^ 1997 United Kingdom	String containing asth* in free text records	80% (75–86)	96% (96–99)	91% (87–94)	94% (93–95)	20.6%

Comparison to an independent database						

Engeland et al,^29^ 2009 Norway	Asthma in MBRN and NorPD	51% (49–52)	98% (98–98)	46% (45–48)		4.20%
Coulter et al,^30^ 1989 United Kingdom	Percentage of people on long term medication and recorded on the register			58%		

Comparison to a questionnaire						

Ward et al,^31^ 2004 United Kingdom	Total of all reviewed patients			89%		5.60%
	Cases without bronchial hyperreactivity			73%		
	Controls with bronchial hyperreactivity			78%		

**Abbreviations:** PPV, positive predictive value; NPV, negative predictive value; NLP, natural language processing; ML, machine learning; MBRN, Medical Birth Registry of Norway; NorPD, Norwegian Prescription Database.

**Table 3:** Quality assessment using QUADAS-2

Study	Risk of bias			
	Patient selection	Index test	Reference standard	Flow and timing
Xi et al,^19^ 2015	☺	?	☺	?
Engelkes et al,^20^ 2014	☺	☺	☹	☺
Afzal et al,^21^ 2013	☹	☺	☺	☺
Dexheimer et al,^22^ 2013	☺	☺	☺	☺
Wu et al,^23^ 2013,2014	☹	☺	?	☺
Kozyrskyj et al,^24^ 2009	☹	☹	☺	☺
Pacheco et al,^25^ 2009	☹	☺	☺	☺
Vollmer et al,^26^ 2004	☹	☺	☺	☺
Donahue et al,^27^ 1997	☺	☹	☹	☺
Premaratne et al,^28^ 1997	☺	☹	☺	☺
Engeland et al,^29^ 2009	☹	☹	☹	☹
Coulter et al,^30^ 1989	☹	☹	☹	?
Ward et al,^31^ 2004	☹	☹	☺	☹

**Note:** Happy face: low risk; sad face: high risk; question mark: unclear risk.
